# Supplementary material for: Apico-basal cell compression regulates Lamin A/C levels in epithelial tissues
Source: Nat Commun. 2021 Mar 25;12:1756. doi: 10.1038/s41467-021-22010-9 (PMC7994818; doi:10.1038/s41467-021-22010-9)
Supplement: Supplementary file 1 — Supplementary Information [file 41467_2021_22010_MOESM1_ESM.pdf]

Apico-basal cell compression regulates Lamin A/C levels in Epithelial tissues

K Venkatesan Iyer[1,3,#,*], Anna Taubenberger[4], Salma Ahmed Zeidan[1], Natalie A. Dye[1,2], Suzanne Eaton[1,2,†], Frank Jülicher[2,3,5,*]

[1]Max Planck Institute of Molecular Cell Biology and Genetics, Pfotenhauerstr. 108, Dresden,Germany
[2]Cluster of Excellence Physics of Life, TU Dresden, 01062 Dresden, Germany
[3]Max Planck Institute for Physics of Complex Systems, Nöthnitzerstr. 38, 01138, Dresden,Germany
[4]Biotechnology Center TU Dresden, Tatzberg 47-49, 01307 Dresden,Germany
[5]Center for Systems Biology Dresden, Pfotenhauerstr.108, Dresden,Germany
[#]Present Address: Mechanical Engineering Department, Indian Institute of Science, 560012, Bangalore, India

Supplementary Information

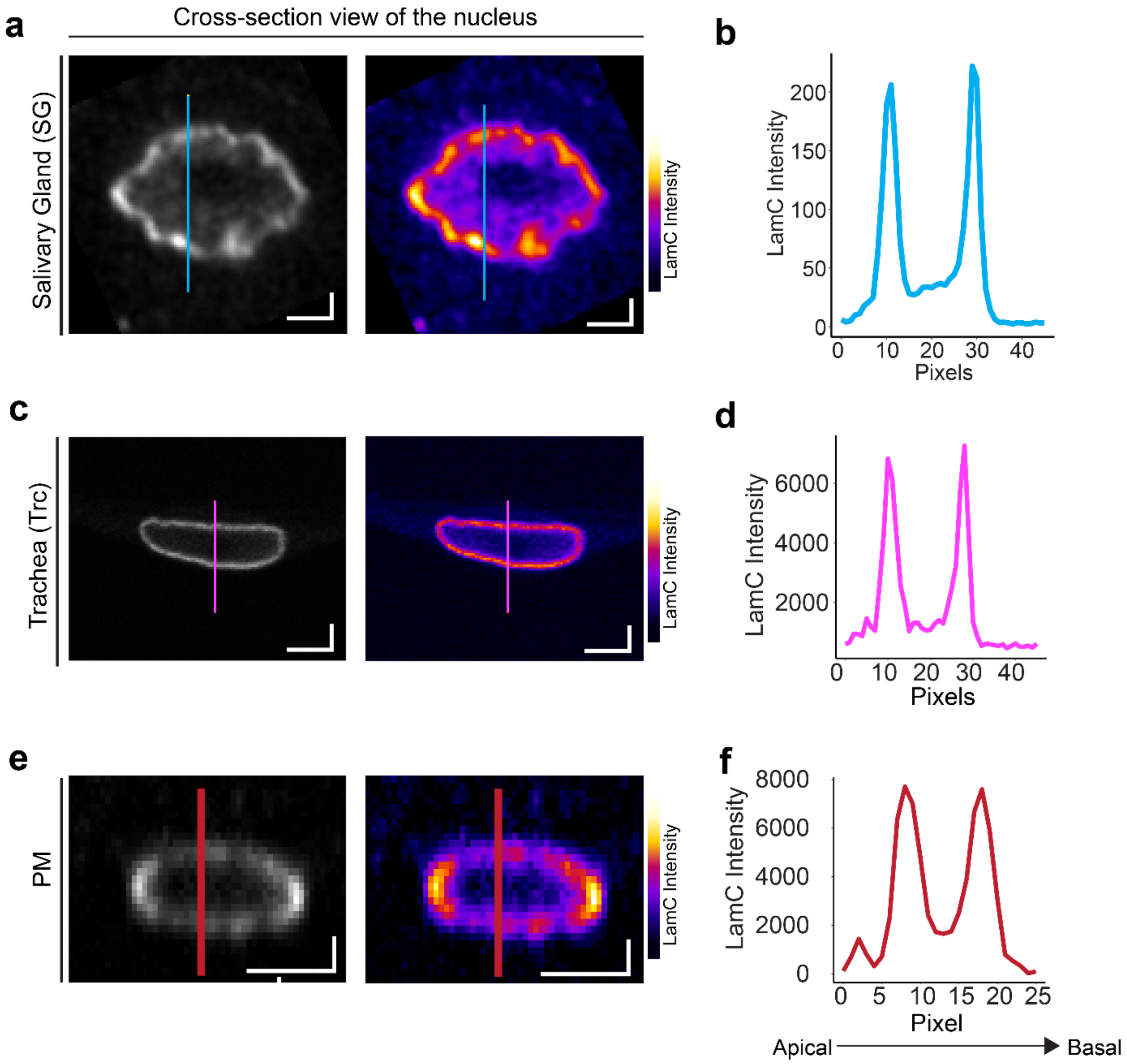

Supplementary Fig 1: LamC is uniformly distributed along the apical and basal axis of the nucleus. (a) Grayscale and color-coded images of cross-section view of the nucleus of Salivary Gland. **(b)** Line profile of LamC along the blue line shown in (a). **(c)** Grayscale and color-coded images of cross section view of the nucleus of Trachea **(d)** Line profile of LamC along the magenta line shown in (c). **(e)** Grayscale and color-coded images of cross-section view of the nucleus of peripodial membrane **(f)** Line profile of LamC along the dark red line shown in (e). Vertical and horizontal scale bar, 5 µm

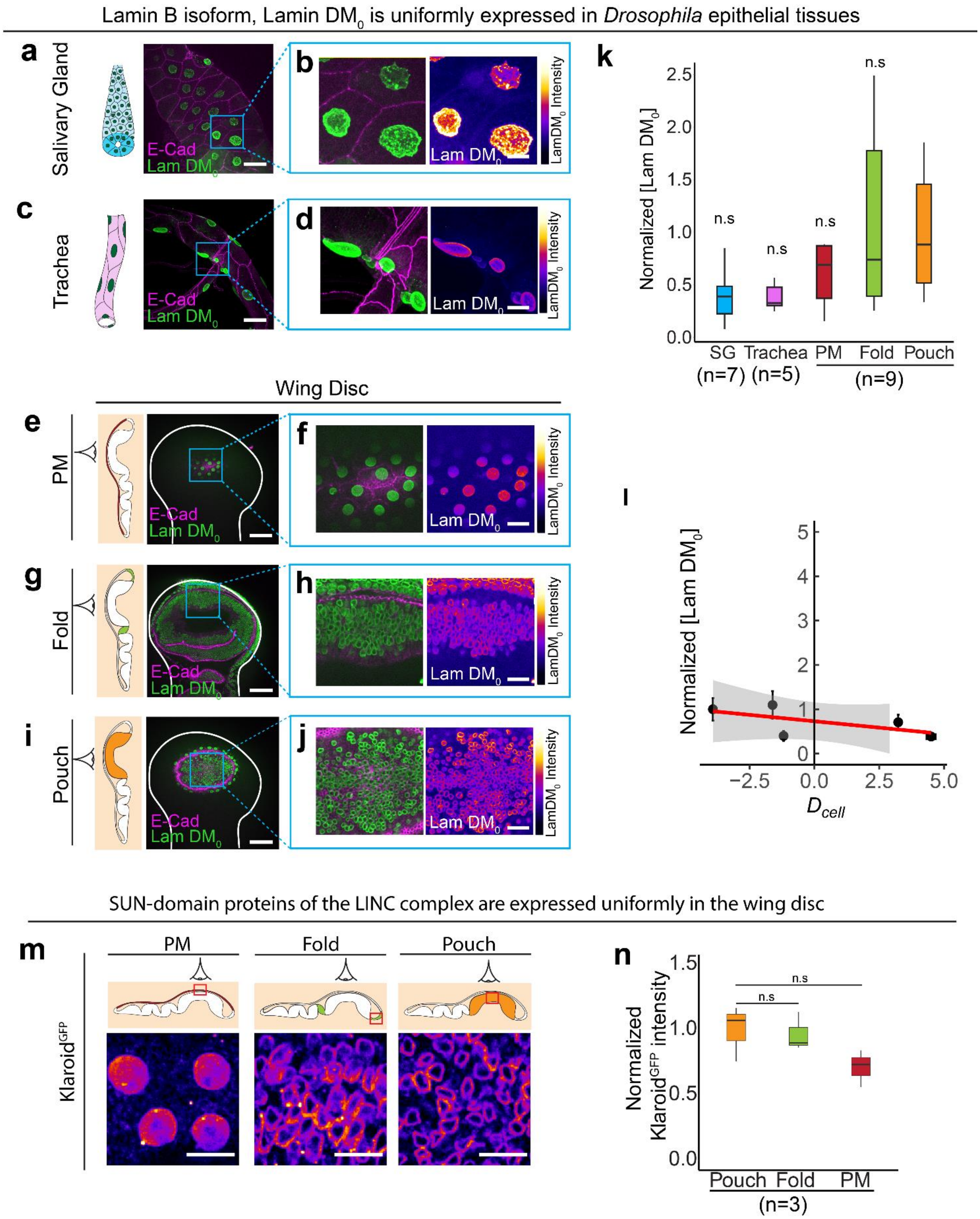

Supplementary Fig. 2: Lamin DM$_0$ is and SUN domain proteins are similarly expressed in epithelial tissues. (a) Image showing E-Cad (magenta) and LamDM$_0$ (green) in salivary gland. **(b)** Enlarged image of the region marked by blue ROI in (a). Left panel shows the merge

of E-Cad and LamDM$_0$. Right panel shows LamDM$_0$ color coded for intensity. **(c)** Image showing E-Cad (magenta) and LamDM$_0$ (green) in trachea. **(d)** Enlarged image of the region marked by blue ROI in (c). Left panel shows the merge of E-Cad and Lam DM$_0$. Right panel shows LamDM$_0$ color coded for intensity. **(e)** Image showing E-Cad (magenta) and LamDM$_0$ (green) in wing disc PM. **(f)** Enlarged image of the region marked by blue ROI in (e). Left panel shows the merge of E-Cad and LamDM$_0$. Right panel shows LamDM$_0$ color coded for intensity. **(g)** Image showing E-Cad (magenta) and LamDM$_0$ (green) in wing disc fold. **(h)** Enlarged image of the region marked by blue ROI in (g). Left panel shows the merge of E-Cad and LamDM$_0$. Right panel shows LamDM$_0$ color coded for intensity. **(i)** Image showing E-Cad (magenta) and LamDM$_0$ (green) in wing disc pouch. **(j))** Enlarged image of the region marked by blue ROI in (i). Left panel shows the merge of E-Cad and LamDM$_0$. Right panel shows LamDM$_0$ color coded for intensity. **(k)** Box plot showing the normalized LamDM$_0$ levels in different *Drosophila* tissues. Normalization is performed with respect to wing disc pouch. The sample *n* biologically independent tissue samples **(l)** Scatter plot between D_{cell} and normalized LamDM$_0$. Data are represented as mean values ± S.E.M. **(m)** Images showing SUN domain protein Klaroid^{GFP} (Koi) in different regions of the wing disc. (**n**) Box-plot showing the normalized Klaroid^{GFP} intensity in different regions of the wing disc. The sample number *n* in graphs represents the number of independent tissues analyzed. In the box-plot, horizontal line represents the median of the data, lower and upper bounds of the box represent the 25th and 75th percentile of data, and the whiskers represent the minimum and maximum of the data. The scattered point on the box represents the actual data points. Scale bar in overview images, 50 µm. Scalebar in enlarged images, 15 µm. Scalebar in (m), 10µm. P-values were estimated by one-way ANOVA. Comparison is shown with wing pouch and n.s represents that the differences are not significant.

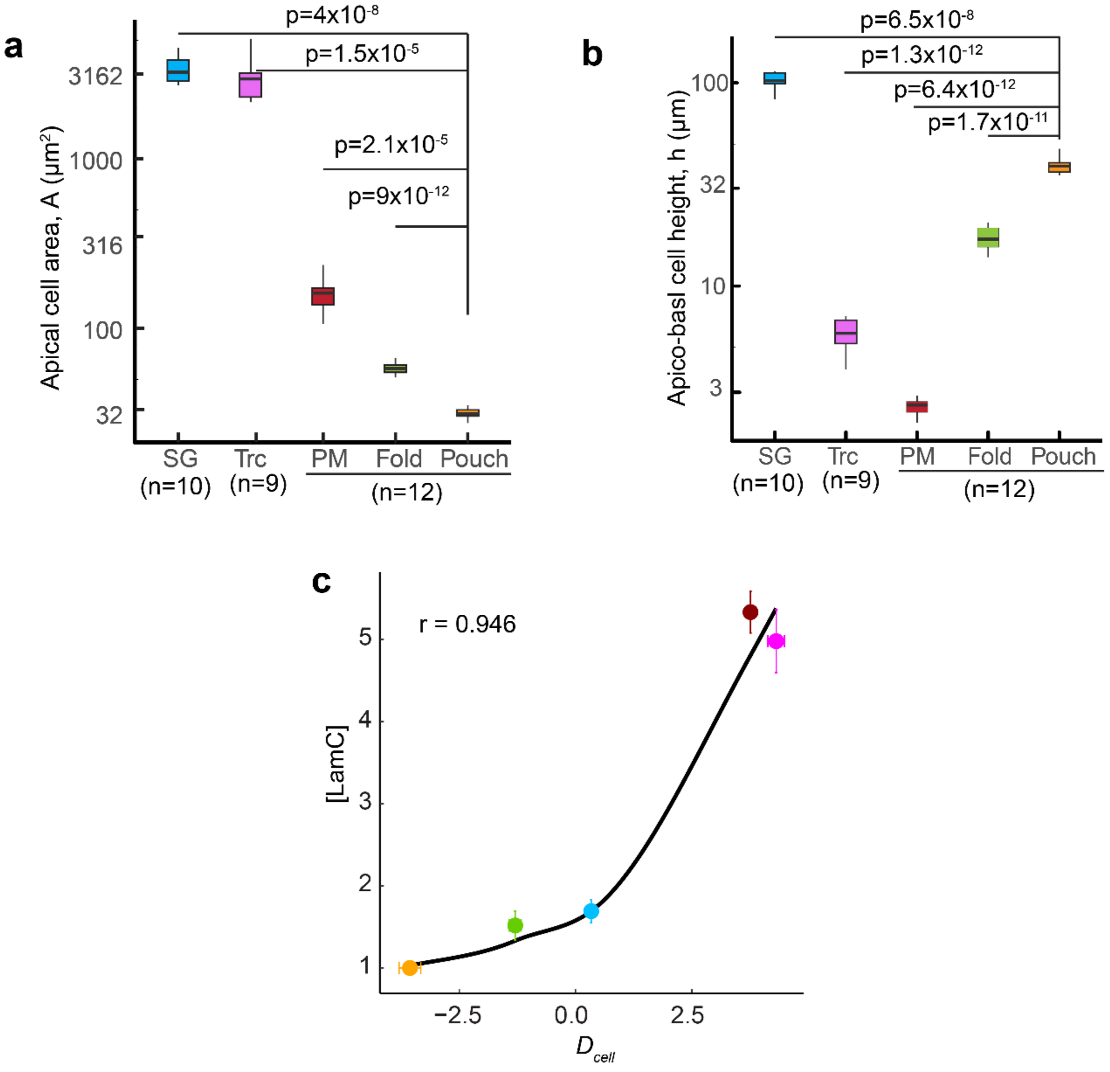

Supplementary Fig. 3: Cell morphology and LamC in Drosophila tissues. (a) Box plot showing apical cell area in *Drosophila* tissues. Y -axis is shown in logscale. **(b)** Box plot showing apico-basal cell height in *Drosophila* tissues. Y-axis is shown in logscale. The sample number n represents the number of tissues analyzed. In the box-plot, horizontal line represents the median of the data, lower and upper bounds of the box represent the 25th and 75th percentile of data, and the whiskers represent the minimum and maximum of the data. The sample number *n* represents the number of biologically independent tissue samples. The scattered point on the box represents the actual data points**. (c)** Scatter plot between D_{cell} and LamC in *Drosophila* tissues. Each point represents average LamC and D_{cell} for *Drosophila* tissues. The value r= 0.946 is the Spearman's correlation coefficient between LamC and D_{cell} . Data are represented as mean values ± S.E.M. P-values are estimated by one-way ANOVA and are represented in comparison to the values for pouch.

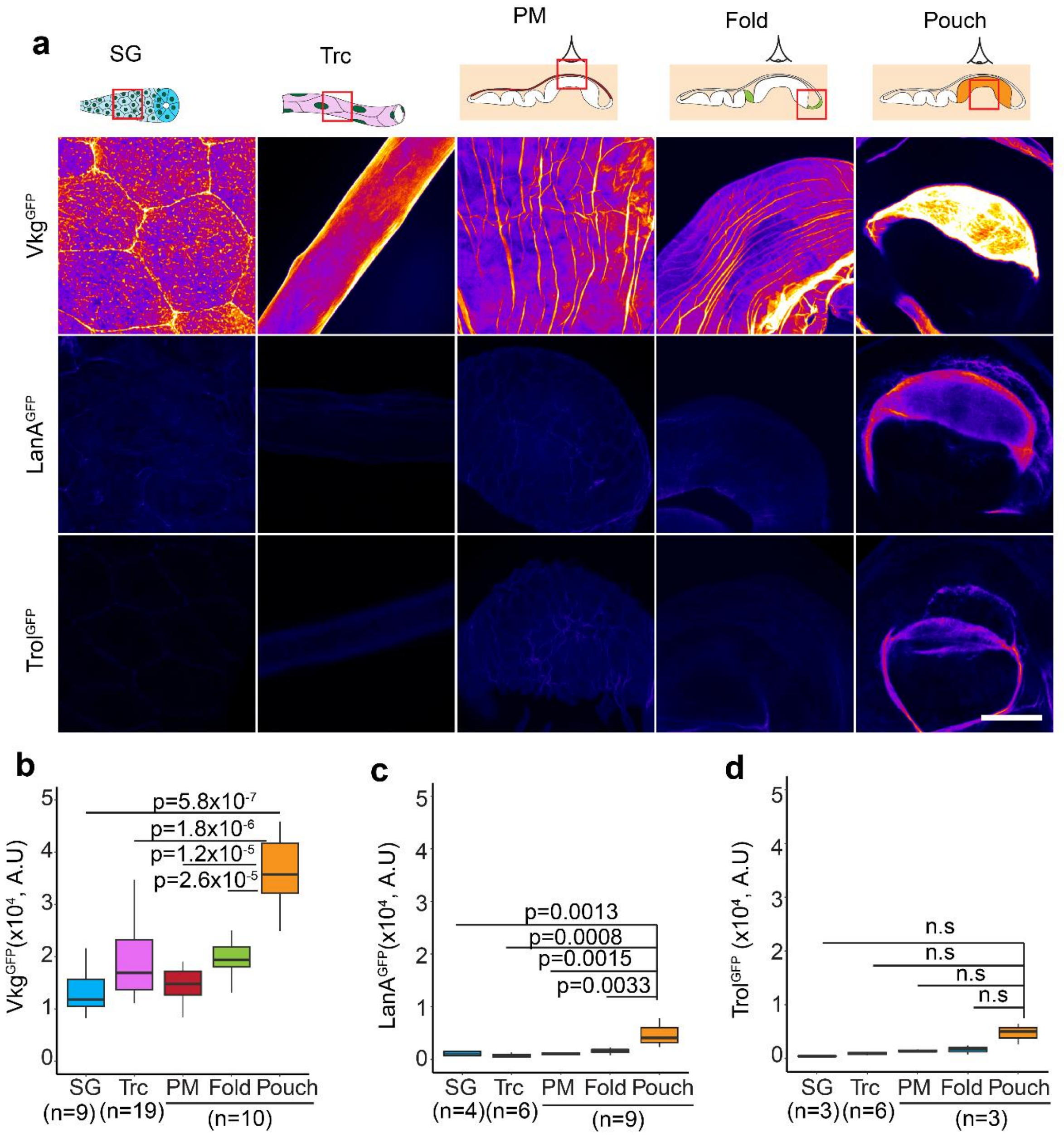

Supplementary Fig 4: Collagen IV has a major contribution to ECM levels. (a) Images showing the levels of Collagen IV (top row), Laminin A (middle row) and Perlecan (bottom row) in Salivary glands (SG), Trachea (Trc), Peripodial Membrane (PM), Fold and Pouch. **(b)** Box plot showing the levels of Collagen IV in different epithelial tissues. **(c)** Box plot showing the levels of Laminin A in different epithelial tissues. **(d)** Box plot showing the levels of Perlecan ($Trol^{GFP}$) in different epithelial tissues. In the box-plot, horizontal line represents the median of the data, lower and upper bounds of the box represent the 25th and 75th percentile of data, and the whiskers represent the minimum and maximum of the data. The scattered point on the box represents the actual data points**.** The sample number *n* shown in the graphs represents the number of independent tissue samples analyzed. P-values are estimated by one-

way ANOVA and are shown in comparison to the pouch and n.s represents that the differences are not significant. Scale bar, 50 µm.

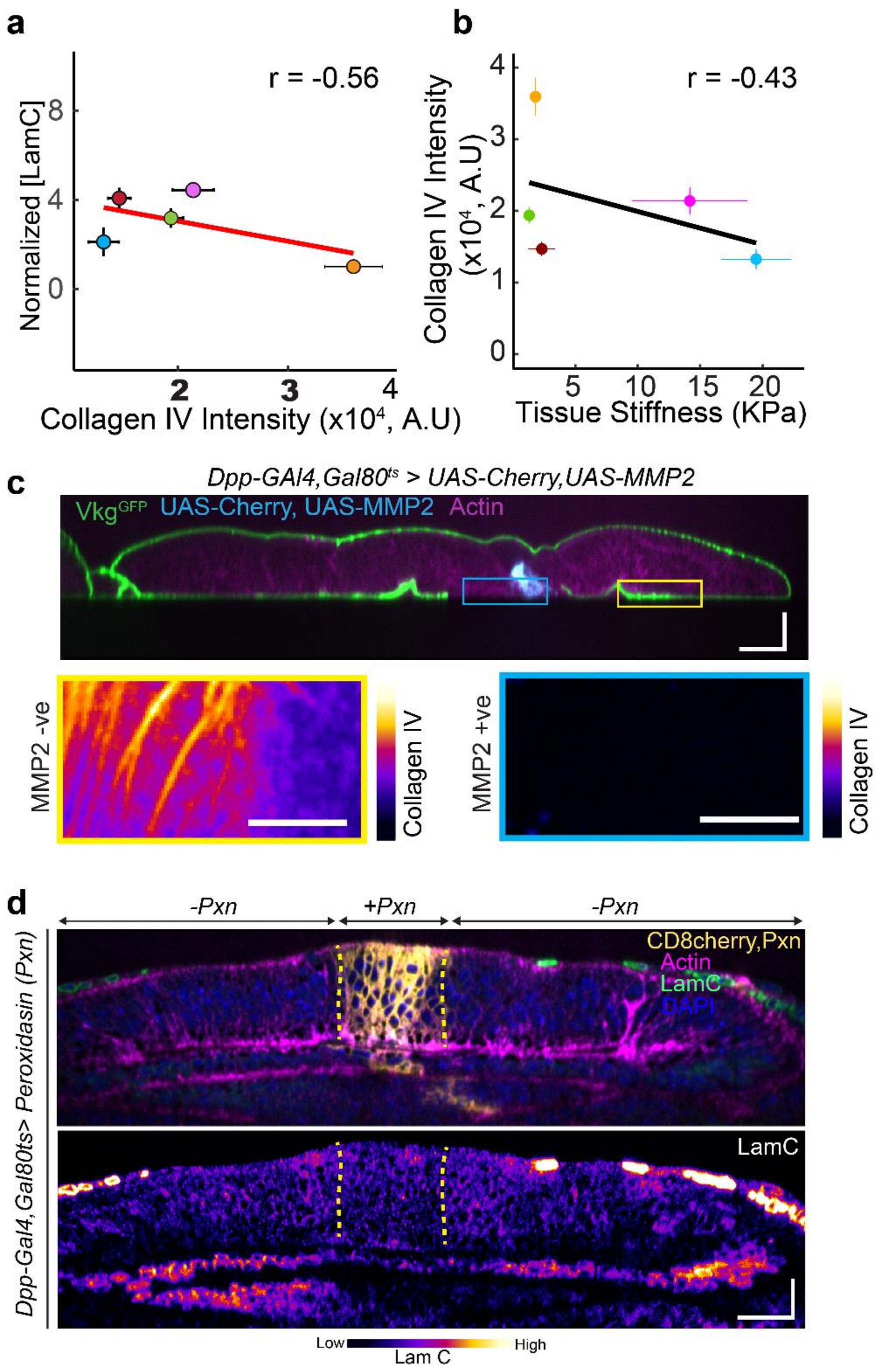

Supplementary Fig. 5: LamC levels in the wing disc are not regulated by ECM levels. (a) Scatter plot between Collagen-IV intensity and LamC levels in epithelial tissues. Each point represents the average value of Collagen IV and LamC levels. The value r in the scatter plot represents the Pearson correlation coefficient between the two quantities. Data are represented as mean values ± S.E.M. **(b)** Scatter plot between Collagen-IV intensity and Tissue stiffness' in epithelial tissues. Each point represents the average value of Collagen IV levels and tissue stiffness. Data are represented as mean values ± S.E.M. **(c)** Top panel: Cross-section view of

the wing disc showing vkg^{GFP} (Collagen IV) in green, actin in magenta and UAS-MMP2,UAS-CD8-Cherry in blue. MMP2 in the wing disc is driven by Dpp-GAL4,Gal80^{ts}. Bottom panel: Enlarged XY sections Collagen IV of the regions marked by blue ROI (MMP2 +ve) and yellow ROI (MMP2 -ve). Images are color coded for Collagen IV intensity. Scale bar in overview image, 25 µm. Scale bar in enlarged images , 15 µm. **(d)** Top panel: Cross-section view of the wing disc showing CD8-Cherry, Pxn labelled in yellow, actin labelled in magenta, LamC labelled in green and Nucleus labelled by DAPI in blue. Bottom panel: Cross-section view of the wing disc showing LamC color coded for intensity. Scale bar, 25 µm.

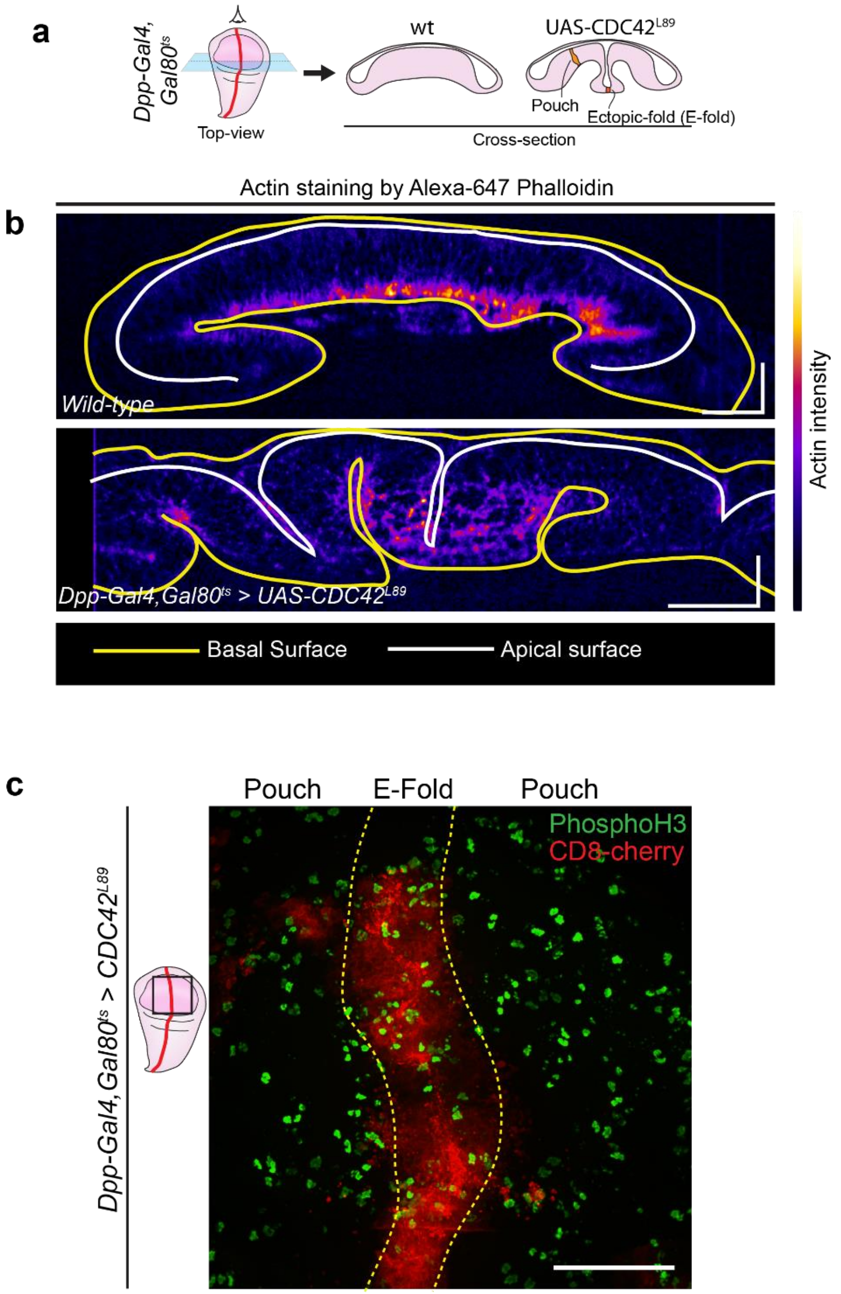

Supplementary Fig. 6: Influence of dominant negative CDC42 overexpression on actin organization and cell proliferation. (a) Schematic showing top view and cross section view of the wing disc expressing dominant negative CDC42 overexpression using Dpp-GAL4. (b) Images showing the color-coded actin intensity in cross-section view of the wild-type wing disc and wing disc expressing CDC42^{L89}. Basal surface of the wing disc is shown in yellow and apical surface of the wing disc is shown in white. Scale bar along horizontal and vertical axes, 25 µm. (c) Image showing proliferating cells marked by phospho-Histone H3 (green) in wing disc expressing dominant negative CDC42. The ectopic fold region is marked by yellow dotted lines. Scale bar, 25 µm.

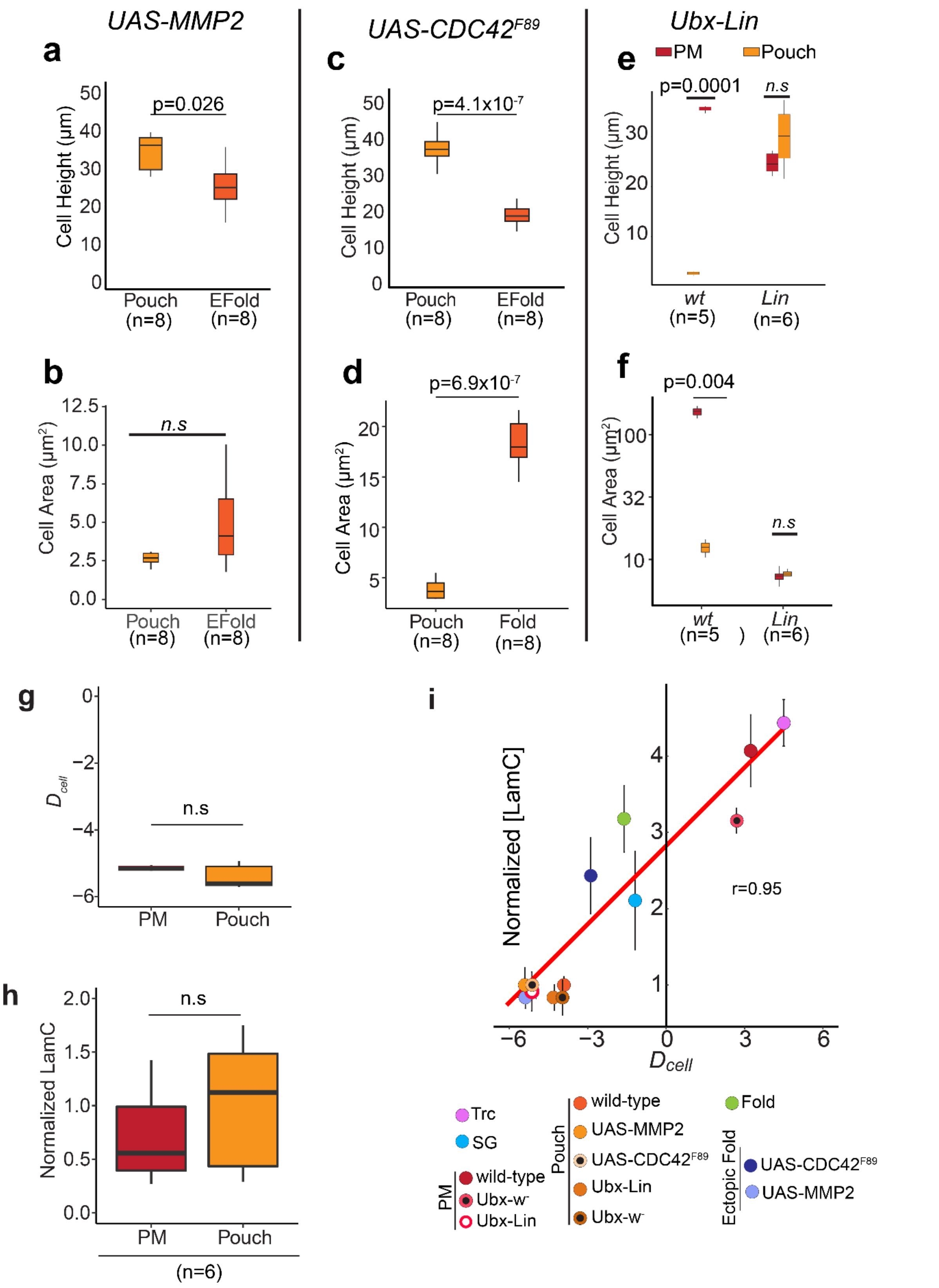

Supplementary Fig. 7: Cell morphology and LamC in *Drosophila* wing disc under different perturbations. (a-b) Cell height (a) and cell area (b) in the pouch (orange) and ectopic fold (dark orange) in wing disc expressing UAS-MMP2 by *Dpp-Gal4,Gal80*^{ts}. **(c-d)** Cell height (c) and cell area (d) in the pouch (orange) and ectopic fold (dark orange) in wing disc expressing UAS-CDC42^{F89} by *Dpp-Gal4,Gal80*^{ts}. **(e-f)** Cell height (e) and cell area (f) in

PM (red) and pouch (orange) regions of the wing disc expressing either w- or *Lin* by Ubx-Gal4. The sample number *n* in the graphs represents the number of tissues analyzed.(**g**) Box plot showing D_{cell} values for PM and pouch in wing discs expression *UAS-Lin*. (**h**) Box plot showing normalized LamC levels for PM and pouch in wing discs expressing *UAS-Lin*. In the box-plot, horizontal line represents the median of the data, lower and upper bounds of the box represent the 25th and 75th percentile of data, and the whiskers represent the minimum and maximum of the data. The scattered point on the box represents the actual data points**.** The sample number *n* in the graphs represents the number of biologically independent tissues analyzed. **(i)** Scatter plot between Normalized LamC levels and D_{cell}. The data includes different perturbations which change D_{cell} in the wing disc. Normalization is done with respect to the *wt* control in the respective experiment. The Pearson Correlation coefficient is 0.95 with FDR of 2×10^{-5}. Data are represented as mean values ± S.E.M. P-values are estimated using two-sided Student's t-test and n.s represents that the differences are not statistically significant.

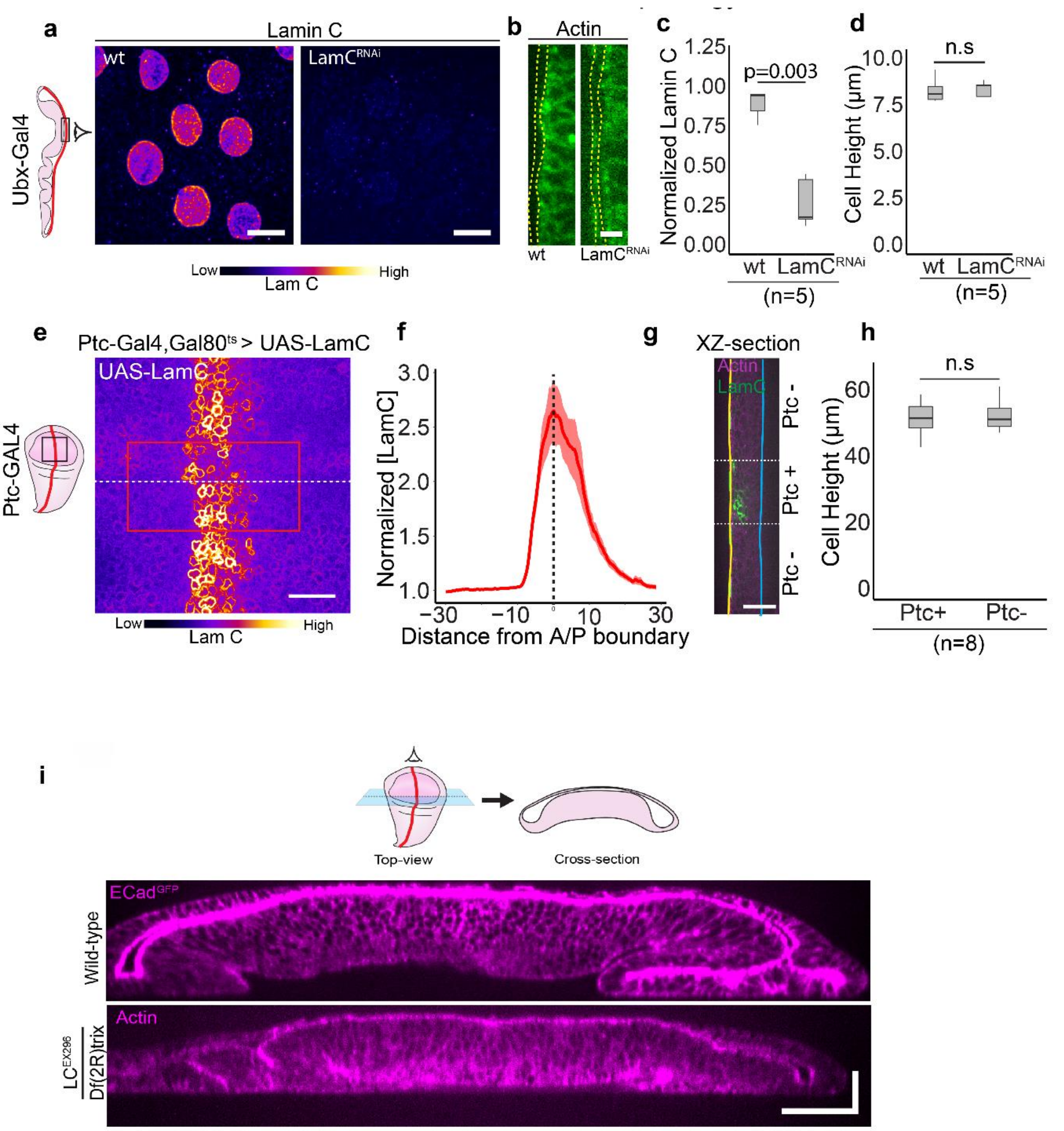

Supplementary Fig. 8: Morphology of wing disc cells is independent of Lamin C levels. **(a)** Images showing LamC in the PM region of the wing discs, in which Ubx-Gal4 either drives w- (left) or UAS-LamC^{RNAi}(right). Scale bar, 10 µm **(b)** Cross-section views of the PM region of the wing disc shown actin (in green) for discs driving w- (left) or UAS-LamC^{RNAi} (right). Yellow dotted lines show the width of the PM layer of the wing disc. Scale bar, 10 µm **(c)** Box plot showing normalized LamC in the PM region of the wing disc. Normalization is performed w.r.t wt. **(d)** Box plot showing cell height in w- and LamC^{RNAi} expressing cells**.** **(e)** Image shows LamC in the pouch region of the wing disc, where LamC is overexpressed by Ptc-GAL4 stripe in temperature controlled manner using Gal80^{ts}. The image is color coded for LamC intensity. Scale bar, 15 µm.**(f)** Normalized LamC intensity profile for the region marked by red ROI in

(e). Data are represented as mean values ± S.E.M. The mean over the region is plotted by the solid red line and the shaded region in red shows the S.E.M. Vertical dotted line marks the A/P boundary. **(g)** XZ section of the wing disc along the dotted line shown in (e). Actin is shown in magenta and LamC is shown in green. The yellow and blue solid lines mark the apical and basal surface of the wing pouch respectively. The two dotted lines marks the region of Ptc expression, where LamC is overexpressed. Scale bar, 50 µm **(h)** Box plot showing cell height in Ptc+ and Ptc- cells. In the box-plot, horizontal line represents the median of the data, lower and upper bounds of the box represent the 25th and 75th percentile of data, and the whiskers represent the minimum and maximum of the data. The scattered point on the box represents the actual data points**.** (**i**) Cross-section view of wild-type wing disc, and wing disc mutant for LamC (LamC^{EX296}/Df(2R)trix. Top panel: wild-type disc with ECadGFP labelled in magenta. Bottom panel: Mutant disc with Actin labelled in magenta. Scale bar along both axes, 25 µm. P-values are estimated using two-sided Student's t-test and n.s represents that differences are not statistically significant.

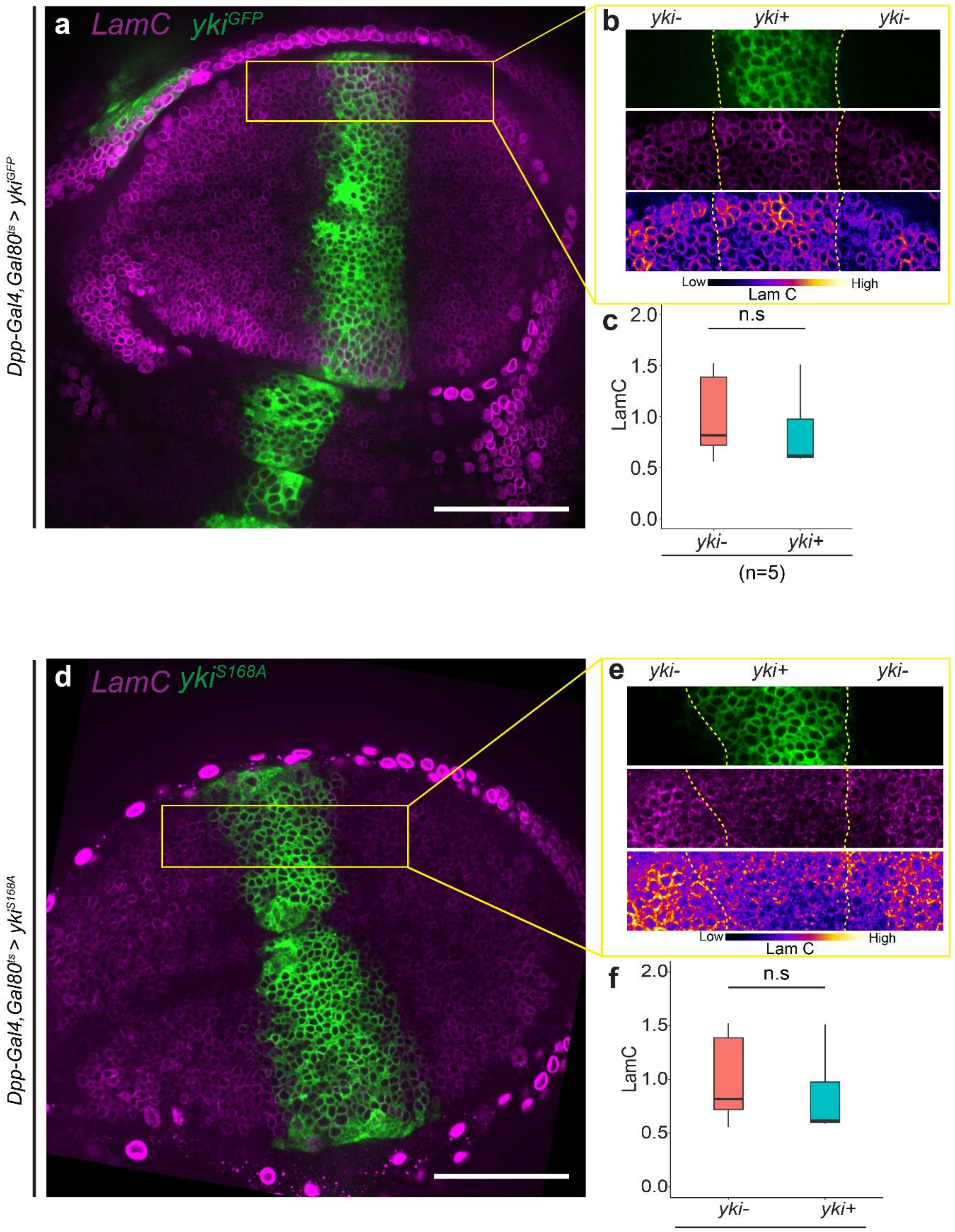

Supplementary Figure 9: Overexpression of Yki does not influence LamC levels in the wing disc. (a) Image showing wing disc expressing UAS-ykiGFP (green) in a stripe of cell driven by Dpp-Gal4. LamC is shown in magenta. **(b)** Enlarged region of the yellow ROI shown in (a). The region of cells expressing yki are marked by a yellow dotted lines. Top panel: Yki, middle panel, LamC, bottom panel: color coded LamC. **(c)** Box-plot showing the levels of LamC in yki expressing (yki+) and yki not expressing (yki-) cells. **(d)** Image showing wing

disc expressing UAS-ykiS168A (marked by CD8-Cherry expressing cells in green) in a stripe of cells driven by Dpp-Gal4. LamC is shown in magenta. **(e)** Enlarged region of the yellow ROI shown in (d). The region of cells expressing yki are marked by a yellow dotted lines. Top panel: Yki, middle panel, LamC, bottom panel: color coded LamC. **(f)** Box-plot showing the levels of LamC in yki expressing (yki+) and yki not expressing (yki-) cells. In the box-plot, horizontal line represents the median of the data, lower and upper bounds of the box represent the 25th and 75th percentile of data, and the whiskers represent the minimum and maximum of the data. The scattered point on the box represents the actual data points**.** P-values are estimated using two-sided Student's t-test and n.s represents that the differences are not significant. Scale bar, 25 µm.

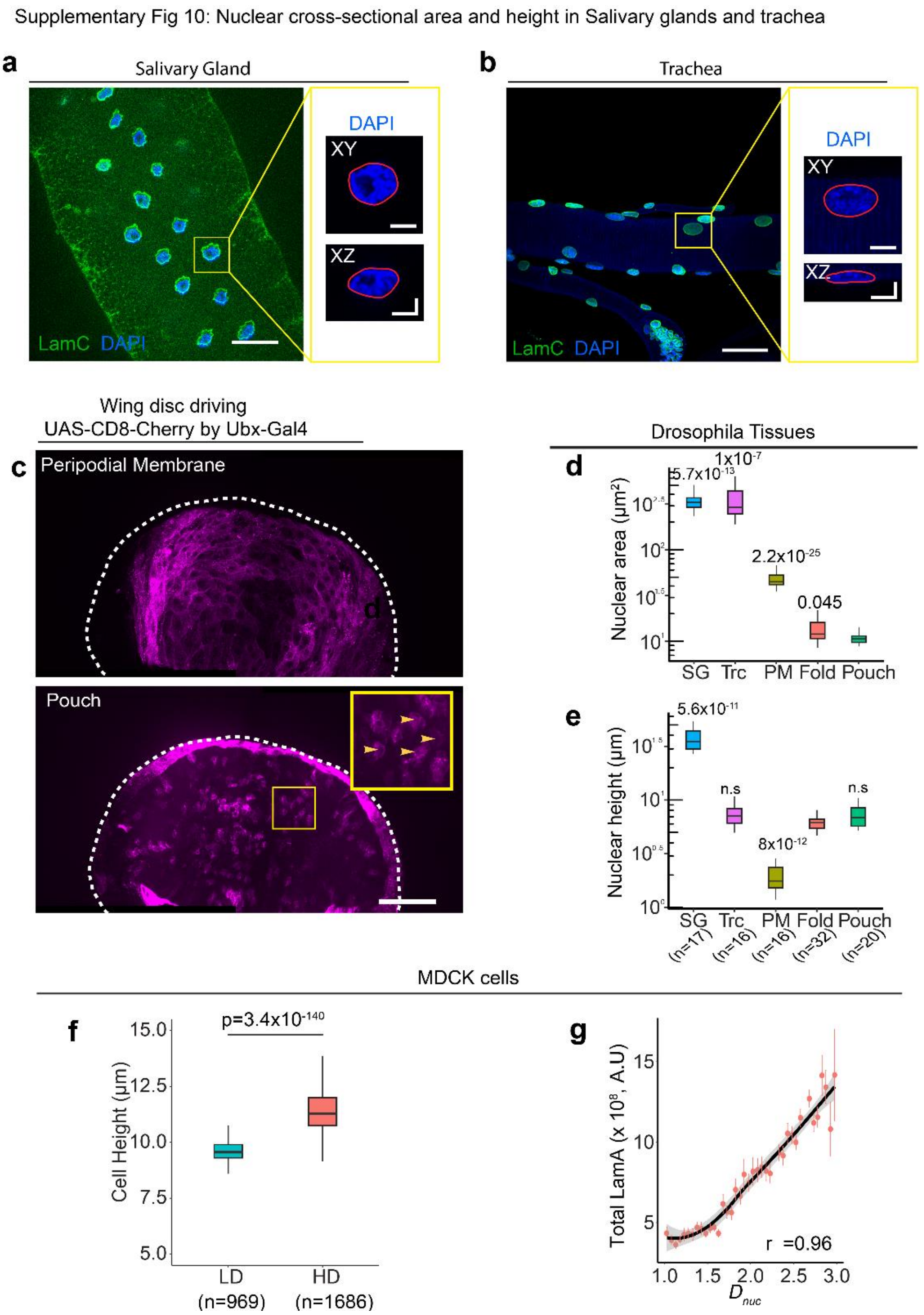

Supplementary Fig. 10: Nuclear morphology in Epithelial tissues. (a) Image showing LamC (green) and DAPI (blue) in Salivary gland. Scale bar, 50 µm**.** Right panel shows the enlarged XY and XZ images of the nucleus marked by Yellow ROI . Nucleus is labelled by DAPI in blue and the nuclear outline is marked by solid red line. Scale bar, 10 µm along both axes. **(b)** Image showing LamC (green) and DAPI (blue) in Trachea. Scale bar, 50 µm. Right panel shows the enlarged XY and XZ images of the nucleus marked by Yellow ROI . Nucleus is labelled by DAPI in blue and the nuclear outline is marked by solid red line. Scale bar, 10 µm along both

axes. **(c)** Wing disc driving UAS-CD8-Cherry by Ubx-Gal4. Left panel shows the PM region and right panel shows the pouch region. Inset image in the right panel is the enlarged image of the region marked by yellow ROI. Arrowheads show individual cells labelled by UAS-CD8-Cherry. Scale bar, 50 µm. **(d)** Box plot showing ox nuclear cross-sectional area in different *Drosophila* tissues. Y axis is shown in logscale. **(e)** Box plot showing nuclear height in different *Drosophila* tissues. Y axis is shown in logscale. (**f**) Box plot showing cell height in low density (cyan) and high density (red) cultures. The sample number *n* represents the number of cells analysed over 3 biologically independent experiments. In the box-plot, horizontal line represents the median of the data, lower and upper bounds of the box represent the 25th and 75th percentile of data, and the whiskers represent the minimum and maximum of the data. The scattered point on the box represents the actual data points**. (g**) Binned scatter plot between D_{nuc} and total Lamin A in MDCK cells. Solid black line shows the LOESS regression fit to the data. Data are represented as mean values ± S.E.M. P-values were estimated using one-way ANOVA and comparison is shown w.r.t the wing pouch and n.s represents that the differences are not statistically significant.

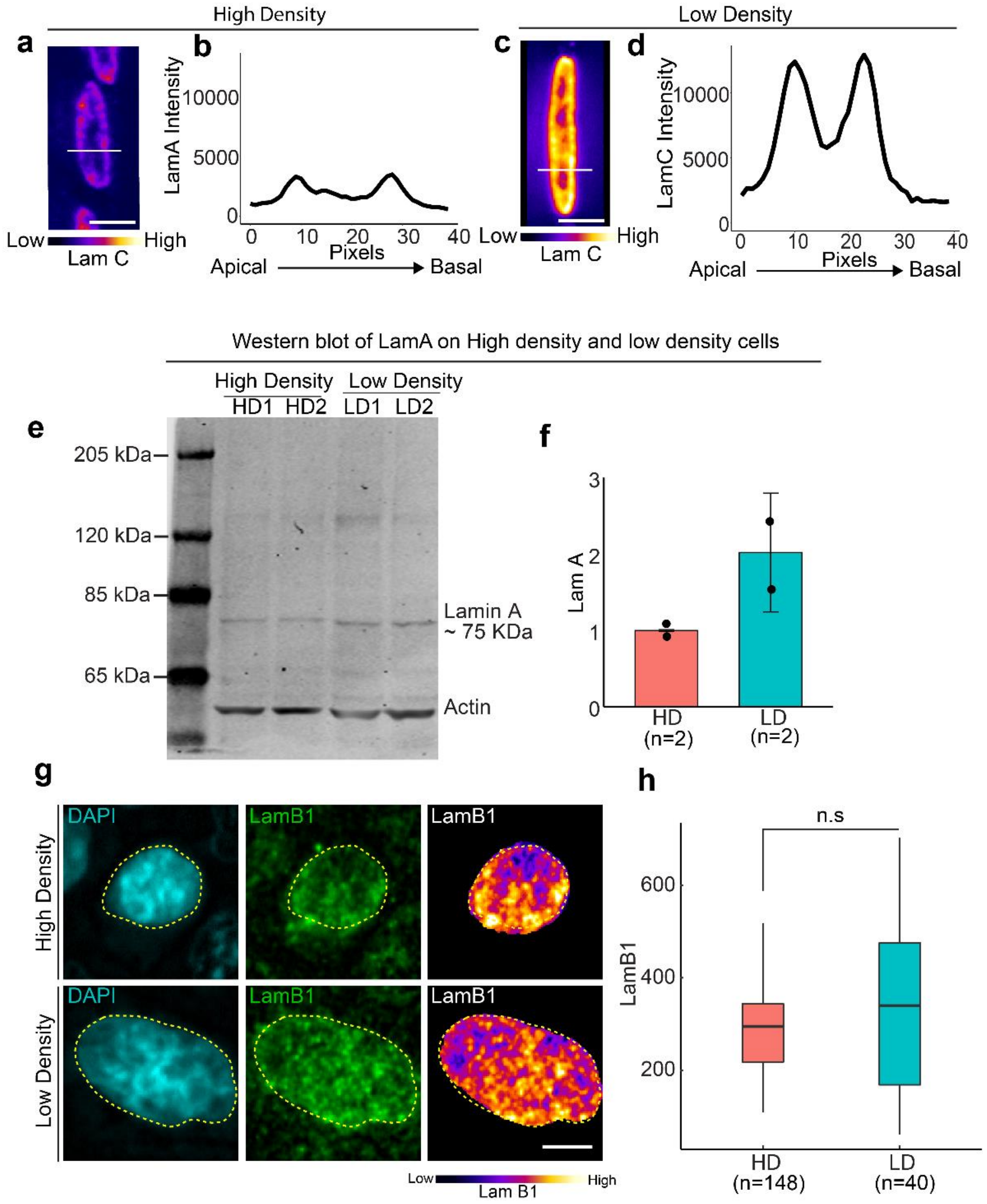

Supplementary Figure 11: LamA and LamB1 staining in MDCK cells. (a) Cross-section view of the nucleus of an HD cell. The image is color-coded for LamA. **(b)** Line profile of LamA in the nucleus along the white line shown in (a). **(c)** Cross-section view of the nucleus of an HD cell. The image is color-coded for LamA. **(d)** Line profile of LamA in the nucleus along the white line shown in (c). Scale bar, 10 µm. **(e)** Image showing the Immunoblot of HD and LD cells in replicates. **(f)** Quantification of the levels of LamA from the immunoblot images. The intensity is normalized to the actin loading control. Data are represented as mean values ± S.E.M. Black points represents the actual data points. **(g)** Images showing DAPI (cyan) and LamB1 (green and color coded) in HD and LD cultures. Scale bar, 5 µm. **(h)** Box plot showing the levels of LamB1 in the nucleus. In the box-plot, horizontal line represents the

median of the data, lower and upper bounds of the box represent the 25th and 75th percentile of data, and the whiskers represent the minimum and maximum of the data. The scattered point on the box represents the actual data points**.** The sample number *n* represents the number of biologically independent experiments. P-values are computed using two-sided Student's t-test and n.s represents that the differences are not significant.

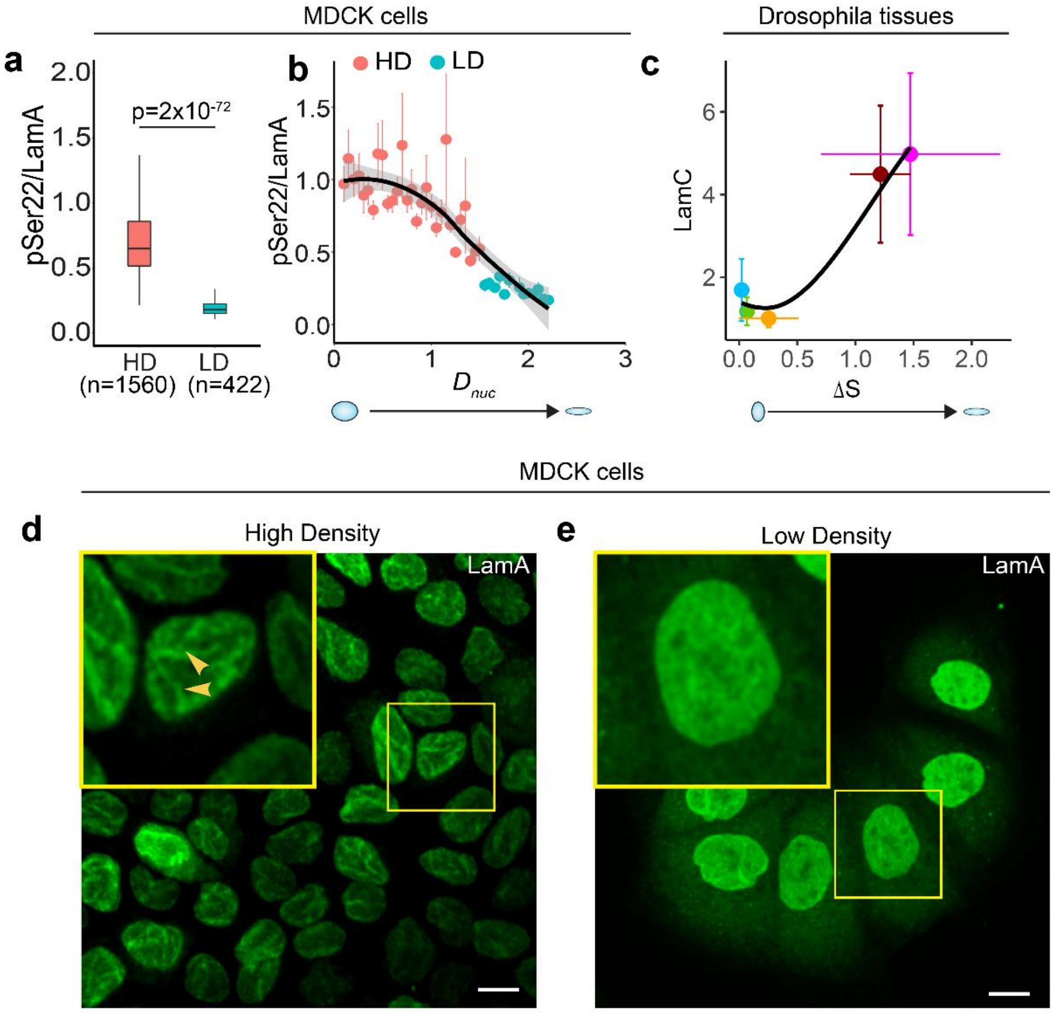

Supplementary Figure 12: Nuclear flattening stretches lamina and alters levels of phosphorylated Lamin A/C. (a) Box plot showing the ratio of pSer22 to LamA in HD and LD cells. In the box-plot, horizontal line represents the median of the data, lower and upper bounds of the box represent the 25th and 75th percentile of data, and the whiskers represent the minimum and maximum of the data. The sample number *n* represents the number of cells analysed over 2 biologically independent experiments. The scattered point on the box represents the actual data points**. (b)** Scatter plot showing the ratio of pSer22 to LamA as a function of D_{nuc} for HD cells (red points) and LD cells (blue points). Solid black line represents the LOESS regression smoothing of the data. Data are represented as mean values ± S.E.M. **(c)** Scatter plot shown LamC levels as a function of the surface area strain in *Drosophila* epithelial tissues – SG (light blue), Trc (magenta), PM (dark red), Fold (light green) and Pouch (orange). Data are represented as mean values ± S.E.M. **(d)** Image showing LamA in high density MDCK cell culture. Inset is the enlarged view of the region marked by yellow ROI. Yellow arrowheads shows undulations in the lamina. **(e)** Image showing LamA in low density MDCK cell culture.

Inset is the enlarged view of the region marked by yellow ROI. Data are represented as mean value ± S.E.M. Scale bar, 10 µm. P-values are estimated using two-sided Student's t-test.
