# Supplementary material for: Apico-basal cell compression regulates Lamin A/C levels in epithelial tissues
Source: Nat Commun. 2021 Mar 25;12:1756. doi: 10.1038/s41467-021-22010-9 (PMC7994818; doi:10.1038/s41467-021-22010-9)
Supplement: Supplementary file 3 — Reporting Summary [file 41467_2021_22010_MOESM3_ESM.pdf]

nature research

Corresponding author(s): Frank Julicher and K. Venkatesan Iyer

Last updated by author(s): Feb 10, 2021

Reporting Summary

Nature Research wishes to improve the reproducibility of the work that we publish. This form provides structure for consistency and transparency in reporting. For further information on Nature Research policies, see our Editorial Policies and the Editorial Policy Checklist.

Statistics

For all statistical analyses, confirm that the following items are present in the figure legend, table legend, main text, or Methods section.

n/a	Confirmed	
☐	☒	The exact sample size (*n*) for each experimental group/condition, given as a discrete number and unit of measurement
☐	☒	A statement on whether measurements were taken from distinct samples or whether the same sample was measured repeatedly
☐	☒	The statistical test(s) used AND whether they are one- or two-sided<br>*Only common tests should be described solely by name; describe more complex techniques in the Methods section.*
☒	☐	A description of all covariates tested
☐	☒	A description of any assumptions or corrections, such as tests of normality and adjustment for multiple comparisons
☐	☒	A full description of the statistical parameters including central tendency (e.g. means) or other basic estimates (e.g. regression coefficient) AND variation (e.g. standard deviation) or associated estimates of uncertainty (e.g. confidence intervals)
☐	☒	For null hypothesis testing, the test statistic (e.g. *F*, *t*, *r*) with confidence intervals, effect sizes, degrees of freedom and *P* value noted<br>*Give P values as exact values whenever suitable.*
☒	☐	For Bayesian analysis, information on the choice of priors and Markov chain Monte Carlo settings
☒	☐	For hierarchical and complex designs, identification of the appropriate level for tests and full reporting of outcomes
☐	☒	Estimates of effect sizes (e.g. Cohen's *d*, Pearson's *r*), indicating how they were calculated

Our web collection on statistics for biologists contains articles on many of the points above.

Software and code

Policy information about availability of computer code

Data collection	Andor iQ software was used to collect data
Data analysis	ImageJ-FIJI was used to prepare microscope images for analysis<br>MATLAB (Mathworks) version R16 was used for anlaysing images<br>AFM data were analysed using JPK Analysis software (JPK DP, JPK Instruments)<br>Western blots were analysed using Image Studio Lite (LI-COR Biotechnology, USA)

For manuscripts utilizing custom algorithms or software that are central to the research but not yet described in published literature, software must be made available to editors and reviewers. We strongly encourage code deposition in a community repository (e.g. GitHub). See the Nature Research guidelines for submitting code & software for further information.

Data

Policy information about availability of data

All manuscripts must include a data availability statement. This statement should provide the following information, where applicable:

- Accession codes, unique identifiers, or web links for publicly available datasets
- A list of figures that have associated raw data
- A description of any restrictions on data availability

The data sets generated and analysed during the current study are available from the corresponding author on reasonable request.

Field-specific reporting

Please select the one below that is the best fit for your research. If you are not sure, read the appropriate sections before making your selection.

☒ Life sciences ☐ Behavioural & social sciences ☐ Ecological, evolutionary & environmental sciences

For a reference copy of the document with all sections, see nature.com/documents/nr-reporting-summary-flat.pdf

Life sciences study design

All studies must disclose on these points even when the disclosure is negative.

Sample size	Sample sizes were chosen such that the mean and standard deviation can be reliably estimated. Thus most of the experiments included 5 or more independent biological replicates.
Data exclusions	No data were excluded
Replication	Replication of the experiments were successful. Experiments were performed independantly atleast 3 times to test replicability
Randomization	This is not relevant for this study as samples were not grouped
Blinding	Blinding is not relevant for this study as the samples were not grouped

Reporting for specific materials, systems and methods

We require information from authors about some types of materials, experimental systems and methods used in many studies. Here, indicate whether each material, system or method listed is relevant to your study. If you are not sure if a list item applies to your research, read the appropriate section before selecting a response.

Materials & experimental systems

n/a	Involved in the study	
☐	☒	Antibodies
☐	☒	Eukaryotic cell lines
☒	☐	Palaeontology and archaeology
☐	☒	Animals and other organisms
☒	☐	Human research participants
☒	☐	Clinical data
☒	☐	Dual use research of concern

Methods

n/a	Involved in the study	
☒	☐	ChIP-seq
☒	☐	Flow cytometry
☒	☐	MRI-based neuroimaging

Antibodies

Antibodies used

Lamin C: obtained from Developmental Studies Hybridoma Bank (DSHB), Cat No. LC28.26
Lamin DM0: Obtained from DSHB, Cat No. ADL67.10
Lamin A: Obtained from Thermo Fischer, Cat No. MA1-06101, Clone Name: 133A2
ZO1: Obtained from Thermo Fischer, Cat No. 40-2200
phospho Ser22 LamA: Obtained from Cell Signaling Technologies, cat No. 2026S
Lam B1: Obtained from Abcam, Cat No. ab8982
Actin: Obtained from Abcan, Cat No. 8227
Polyclonal phospho-Histone H3: obtained from Sigma Aldrich, Cat No. 06-570
Goat anti-mouse Alexa 647: Obtained from Thermo Fischer, Cat No. A28181
Goat anti-mouse Alexa 488: Obtained from Thermo Fischer, Cat No. A-21121
Donkey anti-Rabbit IgG IRdye 680 : Obtained from LiCor, Cat No. 926-68073
Donkey anti-Mouse IRdye 800: Obtained from LiCor, Cat No. 926-32212

Validation

Lamin A: The antibody was verified by relative expression to ensure that the antibody binds to the antigen stated.
ZO-1: This Antibody was verified by Knockdown to ensure that the antibody binds to the antigen stated.

Eukaryotic cell lines

Policy information about cell lines

Cell line source(s)	Madin Darby Canine Kidney cells (MDCK II), Public Health England, cat#00062107
Authentication	The cell line was not authenticated

Mycoplasma contamination	The cell line is tested negative for mycoplasma
Commonly misidentified lines (See ICLAC register)	No

Animals and other organisms

Policy information about studies involving animals; ARRIVE guidelines recommended for reporting animal research

Laboratory animals	Drosophila melanogaster. Following strains were used:<br>w-;DECADGFP : Obtained from Hong Lab<br>w-;UAS-CD8::Cherry;Dpp-GAL4,Gal80ts : Obtained from Dahmann Lab, TU Dresden<br>yw;vkg::GFP and w-;Ptc::Gal4,Gal80ts : Obtained from MPI_CBG Fly Facilty<br>w-;UAS-mmp2 : Obtained from Bloomington Stock Center (#58705)<br>w-;UAS-Lin : Obtained from Bloomington Stock Center(#7074)<br>w-;UAS-CDC42F89 : Obtained from Bloomington Stock Center(#6286)<br>w-;UAS-CDC42L89 : Obtained from Bloomington Stock Center(#6289)<br>w-;UAS-ykiGFP : Obtained from Bloomington Stock Center(#28815)<br>w-;UAS-ykiS168A : Obtained from Bloomington Stock Center(#28818)<br>w-;trol::GFP : Obtained from Bloomington Stock Center(#60214)<br>w-;UAS-Stinger::GFP: : Obtained from Bloomington Stock Center (#84277)<br>w-;Df(2R)trix/CyO : Obtained from Bloomington Stock Center(#1896)<br>w-;Laminin-A::GFP : Obtained from Vienna Drosophila Stock Center (#318155)<br>UAS-LamC: Obtained from the Wallrath Lab<br>w-;LamCEX296/CyO: Obtained from the Wallrath Lab.<br>w-;UAS-LamCRNAi: Obtained from National Institute Genetics (#10119R-1)<br>w-;UAS-MMP2,Vkg::GFP : Generated in this work<br>w-;CD8::Cherry; Ubx-GAL4: Generated in this work
Wild animals	The study did not involve wild animals
Field-collected samples	The study did not involve field collected samples
Ethics oversight	No ethical approval or guidance was required as the study used only Drosophila melanogaster

Note that full information on the approval of the study protocol must also be provided in the manuscript.
